# Diurnal patterns of objectively measured physical activity and sedentary behaviour in older men

**DOI:** 10.1186/s12889-015-1976-y

**Published:** 2015-07-04

**Authors:** Claudio Sartini, S. Goya Wannamethee, Steve Iliffe, Richard W. Morris, Sarah Ash, Lucy Lennon, Peter H. Whincup, Barbara J. Jefferis

**Affiliations:** Department of Primary Care & Population Health, University College London, Rowland Hill Street, NW3 2PF London, UK; UCL Physical Activity Research Group, London, UK; School of Social & Community Medicine, University of Bristol, Canynge Hall, 39 Whatley Rd, BS8 2PS Bristol, UK; Division of Population Health Sciences and Education, St George’s University of London, London, UK

**Keywords:** Physical activity, Light activity, Sedentary behaviour, Cohort study, Older adults, Accelerometer, Health conditions, Within day variations

## Abstract

**Background:**

Physical activity (PA) levels among older adults are generally low and sedentary behaviour (SB) very common; increasing PA and reducing SB levels could have appreciable health benefits. Quantifying PA and SB patterns through the day could help in defining strategies for change. We examined within day variations in PA and SB and whether these varied by demographic factors and health status.

**Methods:**

Men aged 71-91 years participating in an established UK population-based cohort study were invited to wear a GT3x Actigraph accelerometer over the hip for one week in 2010-12. Percentages of time spent in sedentary (SB, <100 counts per minute [CPM]); in light (LIPA, 100-1040 CPM) and in moderate to vigorous PA (MVPA, >1040 CPM) were derived. Multilevel models were used to estimate the associations between demographic factors and health status and SB, LIPA and MVPA.

**Results:**

1455 of 3137 men invited (46.4 %) participated and provided adequate data. Men spent 73 % of the day in SB, 23 % in LIPA and 4.5 % in MVPA (619, 197 and 39 min per day respectively). The percentage of time spent in MVPA was highest in the morning, peaking at 10-11 am (8.4 %), and then declining until the evening, with the exception of a small increase at 2-3 pm. LIPA followed a similar pattern. Conversely, SB levels were lowest in the morning and increased throughout the day, peaking at 9 pm (88 %). Men who were older, did not use active transport, had mobility limitations, were obese, depressed, had more chronic health conditions, and were smokers had lower levels of MVPA. The impacts of older age, obesity, mobility limitations and chronic diseases on LIPA, MVPA and SB were more marked in the morning than in the afternoon and evening.

**Conclusions:**

Levels of MVPA and LIPA are highest in the morning (peak at 10-11 am) and decrease during the day. SB increases through the course of the day to peak in the evening. Interventions to encourage older men to be physically active may need to take account of current PA patterns, aiming to prolong active morning bouts of PA and/or reducing SB in the afternoon and evening hours.

## Background

Physical activity (PA) declines with increasing age [1–3] and older adults, especially the oldest old, are the least active age group in the population [4–6]. PA levels of older adults are generally low and levels of sedentary behaviour (SB) are high [7–9]. To implement effective strategies to increase PA and reduce SB, it is important to understand usual PA and SB patterns. Accelerometers permit objective and accurate assessment of these patterns in population-based studies and, of special consideration for older adults, reduce the impact of recall bias (over or under reporting), participants memory loss or cognitive impairment [10]. Accelerometers can give insight into how activity levels vary over the course of the day. However, to date there is very little evidence on how PA and SB are structured throughout the day among older people. Existing studies reporting on how activity varies throughout the course of the day have been small [4, 6–8, 11] or mainly have focussed on global measures of activity as CPM and MVPA without considering the wider range of outcomes including different activity intensities as LIPA, SB and duration of bouts of activity [2]. Existing studies suggest that that older adults were more active in the mornings than during afternoons and evenings [2, 4, 6–8, 11], and this may have implications for strategies aim to increase PA on daily basis.

The aim of this study is to investigate diurnal variations in objectively measured LIPA, MVPA and SB in older men, in a much larger study population than previously investigated. We use data from the British Regional Heart Study, an established population-based cohort of community-dwelling older men. The second aim is to determine the extent to which the diurnal variation in PA and SB is modified by key demographic and health status variables including age, body mass index, social class, health conditions (depression, vision problems, and chronic diseases), mobility limitations and social isolation. In a subsidiary descriptive analysis we explore diurnal variation in long bouts of SB (≥60 min) and MVPA bouts of at least 10 min, because these are an important component of UK national PA guidelines [12].

## Methods

### Participants

The British Regional Heart Study (BRHS) is a prospective cohort of 7735 men recruited from a single local primary care centre in 24 British towns in 1978-80 (age 40-59 years). In 2010-2012, all surviving cohort members resident in the UK (n = 3137) were invited to attend a further physical examination including measurements of weight and height and to participate in a study of objectively measured physical activity. The National Research Ethics Service (NRES) Committee for London provided ethical approval. Participants provided informed written consent to the investigation, which was performed in accordance with the Declaration of Helsinki [13].

### Objective physical activity assessment

#### Procedures for distribution and wearing

Participants were invited to attend an assessment by study nurses at their local primary care centre. All men who attended were asked to wear an Actigraph GT3x accelerometer (Pensacola, Florida) over the right hip on an elasticated belt for 7 days, during waking hours, removing it for bathing, swimming or showering and returning the device by post.

#### Data processing

Actigraph accelerometers record “counts” and steps, which both depend upon the frequency and intensity of the raw acceleration [14]. Accelerometer data were processed using standard methods, as described previously [9]. In brief, raw data from movements registering on the vertical axis were integrated into 60 s epochs; therefore counts per minute (CPM) were derived. Non-wear time was identified and excluded using the R package “Physical Activity” [15], based on (i) periods of continuous zero activity lasting more than 90 min or (ii) periods of zero activity lasting more than 90 min broken only by non-zero counts lasting up to 2 min, provided no activity counts were detected during both the 30 min before and after that interval [9]. Valid wear days were defined as ≥600 min wear time, and participants with at least 3 valid days were included in analyses, a conventional requirement for estimating usual PA level [16].

#### Derived variables

The number of minutes per day spent in SB, LIPA and MVPA was categorised using count-based intensity threshold values of counts per minute developed for older adults [9, 11, 17]: <100 CPM for sedentary behaviour (<1.5 Metabolic Equivalent of Task, MET), 100-1040 for light activity (1.5-3 MET) and >1040 for MVPA,(≥3 MET). The cut-point of 1040 CPM was calibrated to identify moderate intensity activities (≥3 MET) in a sample of older adults [11], but we also investigated the more widely used cut-point of 1952 CPM which was calibrated to identify moderate intensity activities (≥3 MET) in middle-aged adults [17]. Two further summary measures of SB and MVPA were calculated: number of sedentary bouts of at least 1 h (a period of 60 or more consecutive minutes where the accelerometer registers <100 CPM) and MVPA bouts of at least 10 min (a period of 10 or more consecutive minutes where the accelerometer registers more than 1040 CPM).

#### Log diary and questionnaire data

Participants completed a log diary, detailing when the accelerometer was put on and taken off during the seven days of wear. During the first 3 days the men were also asked to report the type of activity (*e.g.* housework, gardening, preparing meals, watching TV) that they did during each hour of the day. Participants’ log diaries were checked and matched against accelerometer data to verify the date on which they started wearing the accelerometer. Age and season were derived from the first wear day. Season was categorised as summer (Jun-Aug), autumn (Sep-Oct), winter (Nov-Feb), and spring (Mar-May). Men were asked “do you have any difficulties getting about outdoors?” which was grouped as “none”, “slight” and “moderate, severe and unable to do”. Men reported a medical diagnosis of any of the following chronic conditions; heart attack, heart failure, angina, diabetes, stroke, osteoporosis, claudication, Parkinson’s disease and chronic kidney disease. Men were classified as having vision problems if they had one or more of glaucoma, macular degeneration or cataract, as in advanced age these are primary causes of visual dysfunction [18, 19]. Men scoring > =2 on the 4-item Geriatric Depression score were classified as depressed [20]. Cigarette smoking was self-reported. Participants completed the Lubben scale of social isolation which asks about interactions with family members and with friends, men scoring <12 were classed as at risk of social isolation [21]. Participants reported which forms of transport they used regularly (car, public transport, dial a ride, walk or cycle), those who reported regular walking or cycling were classified as using active transport. The social class classification was based on the longest-held occupation of subjects reported at study entry in 1978–1980. Participants’ occupations were grouped as non-manual or manual.

#### Statistical methods

Analyses were carried out using STATA/SE 13 [22] and MLwiN Version 2.02 [23]. To give a general overview of the within day variation of total PA, counts per minute and steps were plotted against hour of day. The main outcome variables were the proportions (percentages) of the day spent in (1) sedentary behaviour, (2) light PA and (3) moderate to vigorous PA. Each outcome was calculated according to hour of the day, with the number of minutes that the accelerometer was worn in that hour used as the denominator. Due to sparse data in early morning and late evening, we examined the mean activity counts per hour between 7.00 am and 10.59 pm. Only hours with ≥ 45 valid wear minutes were included. A first descriptive analysis was undertaken: for 1329 men with complete data, the percentage of time spent in SB, LIPA and MVPA was plotted against hour of day. In order to explore whether the diurnal patterns were modified by selected variables, the data were stratified by (i) age group (<75, 75-79, ≥80 years) (ii) mobility limitations (none, slight, moderate/severe/unable to do) (iii) number of chronic conditions (none, 1-2, ≥3) (iv) BMI category (<25, 25-30 and ≥30 kg/m^2^) (v) depression (depressed vs not) (vi) smoking status (current smoker vs not) (vii) social isolation (at risk of isolation vs not at risk) (viii) social class (manual vs non-manual) (ix) use of public transport (walk/cycle vs car/public transport) (x) vision problems (yes vs no) (xi) season (winter vs summer) and (xii) weekend vs weekday.

The distributions of each outcome were investigated: percentage of MVPA distribution was highly positively skewed as reported in previous studies [24, 25]. MVPA data were highly over-dispersed with variance 5 to 6 times higher than the means within each period of the day, so negative binomial model were used to investigate which factors were related to the percentage of time spent in MVPA and the results were reported as rate ratios (RRs) [26]. A RR is as multiplicative factor: any deviation from 1 indicates a percent difference in the outcome relative to the respective reference category (baseline) in the exposure variable. Linear multilevel regression models were used to investigate which factors were related to the percentage of time spent in LIPA and SB (normally distributed). Beta coefficients were reported to estimate the difference in time spent in SB and LIPA between the categories of each explanatory variable against the reference. In all multilevel models Level 1 was period of the day [morning (7 am-12.59 pm), afternoon (1 pm-6.59 pm) and evening (7 pm-10.59 pm)] and Level 2 was the individual. Each period (morning, afternoon and evening) had a minimum of 2 valid hours of wear time.

Two level random-intercept and random-slope models were used with adjustments for age, season, region and part of the day, and one additional covariable at a time (mobility limitations, number of chronic conditions, BMI, depression, smoking status, social isolation, social class, use of public transport, and vision problems). Next, fully adjusted models were run using all explanatory variables together. The random slope in the models allowed us to test the hypothesis that the changes in PA levels over the day varied between different men. The estimated slopes over the course of day were reported as mean differences between afternoon and evening vs morning (baseline).

For variables which were significantly associated with sedentary behaviour, light and MVPA levels, interactions were fitted to test whether the associations differed according to period of the day (morning, afternoon, evening). An overall Wald test for interaction between the categories of those explanatory variables and period of the day (morning, afternoon and evening) was used.

## Results

**Fig. 1:**
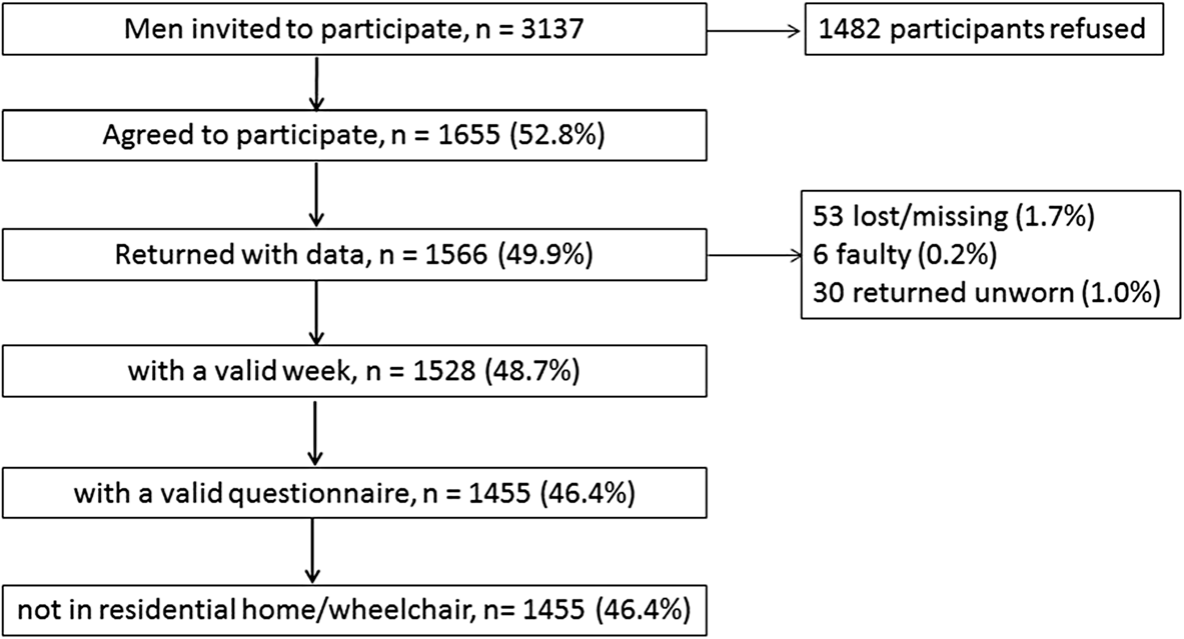
Recruitment flow chart and identification of the eligible population of men

The recruitment flow chart and the inclusion criteria used are presented in Fig. 1. Among 3137 surviving men, 1655 (52.8 % response) agreed to participate and 1455 men (46.4 % response) with a mean age of 78.5 years (range 71-93) provided both physical activity and questionnaire information. All these men were independently mobile and community dwelling. The characteristics of the study participants are shown in Table 1. Men who agreed to participate were younger and 10 years previously had a lower BMI compared to men who did not participate. Participants took on average 4827 steps per day and spent 72.6 % of the day in SB, 22.9 % in light activity and 4.5 % in MVPA (619, 197 and 39 min per day respectively). Participants had a mean of 6.5 (SD = 1.2) valid days of accelerometer wear. 1329/1455 men (91.3 %) had complete data on all covariates, and the same patterns of associations with characteristics in Table 1 are seen in the reduced sample. From this point forward all results refer to the 1329 men (complete case analysis).

**Table 1:** Characteristics of men who met the inclusion criteria for the study and men who did not accept the invitation to participate

	Men who met inclusion criteria for the study	Men who did not meet the inclusion criteria	*p*-value
N	1455	1682	
*Demographic and background characteristics*			
Age, mean (SD)	78.5(4.6)	80.1 (5.2)	<0.001
Region, n(%)			0.031
South	526(36.2)	525(31.2)	
Midlands	217(14.9)	267(15.2)	
North	569(39.1)	701(42.6)	
Scotland	143(9.8)	189(10.9)	
Social class (manual), n(%)	666(45.7)	953 (56.7)	<0.001
*Physical Health*			
BMI, mean (SD)	27.1(3.8)		
BMI 10 years earlier, mean (SD)	26.7(3.3)	27.2(3.8)	<0.001
Number of Chronic conditions, n(%)			
None	674(46.5)		
1-2	668(46.0)		
3+	108(7.5)		
Mobility limitations outdoors, n(%)			
None	915(64.6)		
Slight limitations	264(18.6)		
Moderate/severe difficulty/unable to do	237(16.7)		
Vision Problems (none), n(%)	992(68.2)		
*Mental health and wellbeing*			
Social isolation, (isolated), n(%)^a^	257(17.8)		
Geriatric Depression Scale, (depressed), n(%)^b^	316(22.1)		
*Behaviours*			
Mode of transport used regularly (cycle/walk), n(%)	923(63.4)		
Smoking status (cigarettes), smoker	46(3.2)		
Smoking status 10 years earlier (cigarettes), smoker	97(7.2)	160(12.9)	<0.001
*PA levels/day, mean (IC 95 %)*			
Counts/min (CPM)^c^	186(180,191)		
Steps^c^	4827(4698,4956)		
Percent wear time in SB per day^c,d^	72.6(72.1,73.0)		
Percent wear time in LIPA per day^c,e^	22.9(22.6,23.3)		
Percent wear time in MVPA 1+ per day^e,f^	4.5(4.3,4.7)		
Minutes in SB per day^c,d^	619(615,623)		
Minutes in LIPA per day^c,e^	197(194,200)		
Minutes in MVPA 1+ per day^c,f^	39(37,41)		
Wear time^g^	853(850,857)		
Number of valid days, mean (SD)	6.7(0.8)		

^a^Lubben scale, isolated <12

^b^Geriatric Depression Scale, depressed >2

^c^Means adjusted for age, day order, wear time, season of accelerometer wear and region of residence

^d^Sedentary Behaviour (SB) is at least one minute where the accelerometer registers values <100 CPM

^e^Light physical activity (LIPA) is at least one minute where the accelerometer registers values between 100-1040 CPM

^f^Moderate to vigorous physical activity (MVPA) 1+ is at least one minute where the accelerometer registers values over 1040 CPM

^g^Means are adjusted for age, day order, season of accelerometer wear and region of residence

### Diurnal patterns of PA and SB

**Fig. 2:**
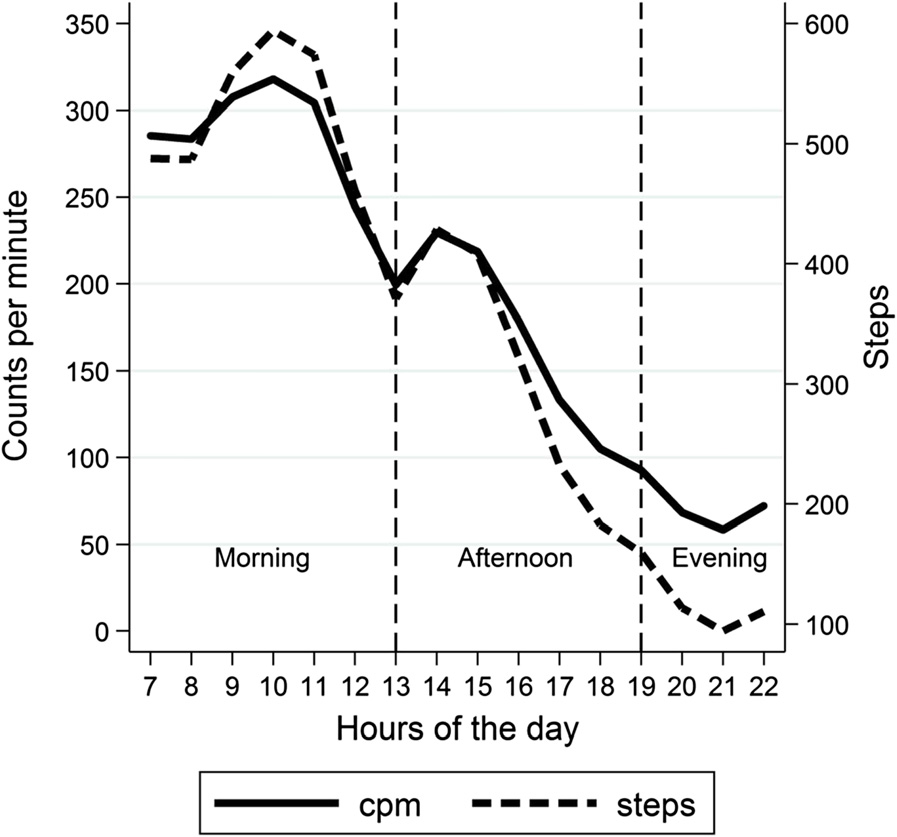
Mean accelerometer counts per minute (CPM) and steps according to hour of day in 1329 men aged 71-93 years

**Fig. 3:**
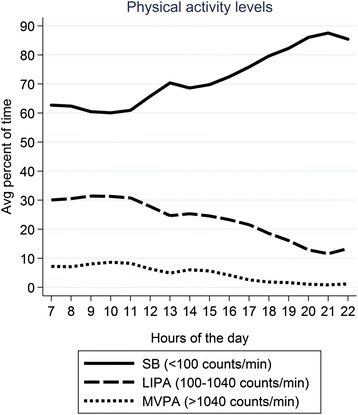
Percentage of total wear time spent in SB, LIPA, and MVPA according to hour of day in 1329 men aged 71-93 years

The intensity of PA indexed by both accelerometer CPM and the number of steps peaked around 10 am and then declined until a small further increase at 2 pm followed by a long decline until 9 pm and then a small increase after 10 pm (Fig. 2). Figure 3 shows the average percentage of time spent in PA of different intensities (SB, LIPA and MVPA) for each hour of the day. LIPA and MVPA were positively correlated within each hour: r = 0.21 (p < 0.001), and conversely, hourly SB was negatively correlated with LIPA (r = -0.88, p < 0.001) and with MVPA (r = -0.64, p < 0.001).

The pattern in daily variation of MVPA and LIPA closely followed the pattern observed for CPM. At around 10 am, the proportions of each hour spent in LIPA and MVPA peaked (at approximately 30 % and 8 % of all activity respectively) and then declined until 1 pm, followed by a slight increase in the afternoon around 2-3 pm and then a long decline until around 9 pm, when light activity accounted for only approximately 10 % and MVPA 1 % of each hour. Conversely, SB levels increased throughout the morning, with a steeper increase before 1 pm and then a small dip around 3 pm, followed by a slow increase to a peak of over 80 % spent in SB between 8-9 pm, followed by a slight decline after 9 pm (Fig. 3). Among men who completed log diaries, commonly reported activities around 11 am were gardening, shopping, moderate housework and do-it-yourself (DIY). Similar activities were reported around 3 pm, although more men reported gardening and fewer men reported housework in the afternoon.

**Fig. 4:**
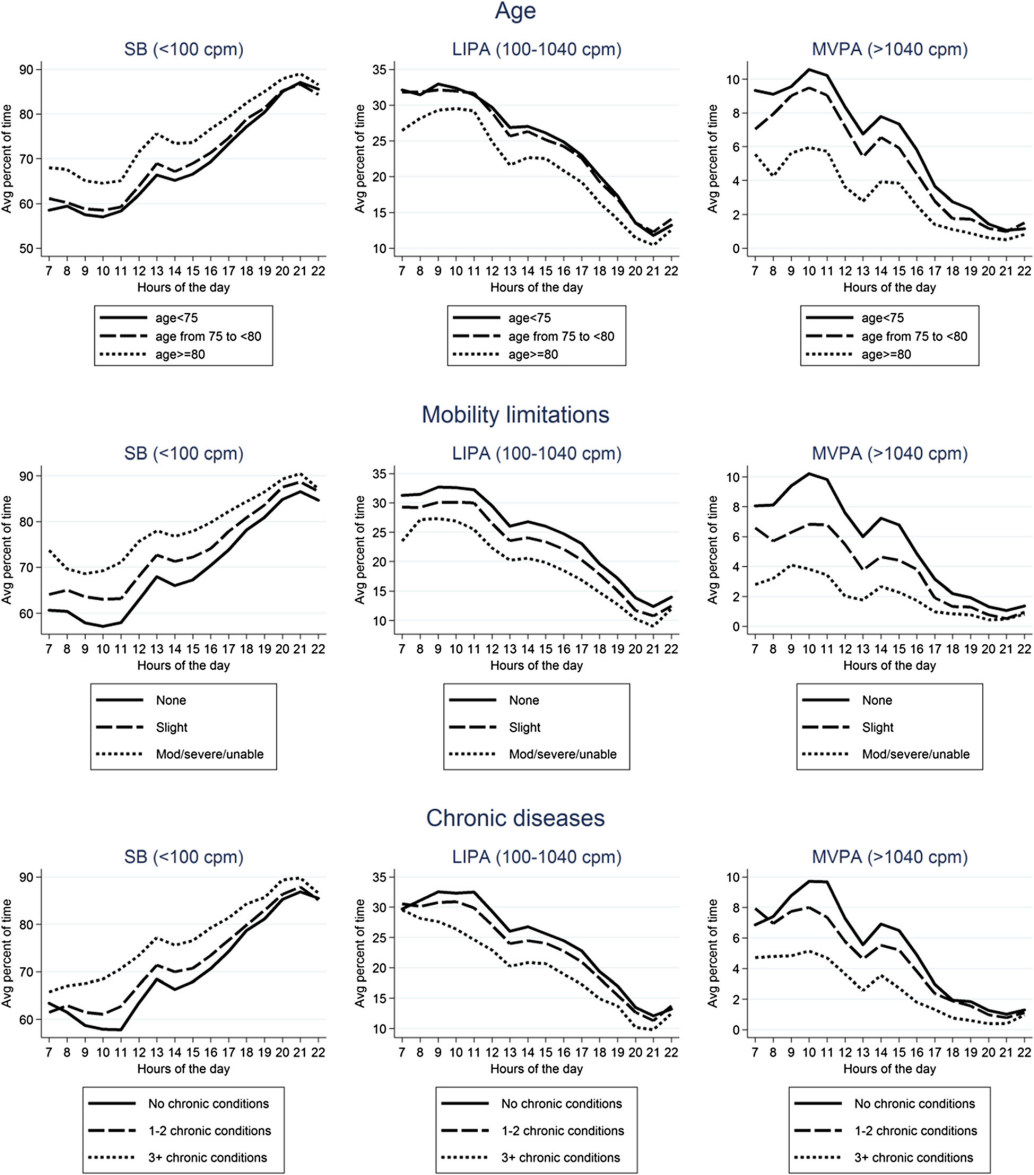
Plots from raw data: percentage of total wear time spent in SB, LIPA, and MVPA according to hour of day, stratified by age group, mobility limitations and chronic conditions

**Fig. 5:**
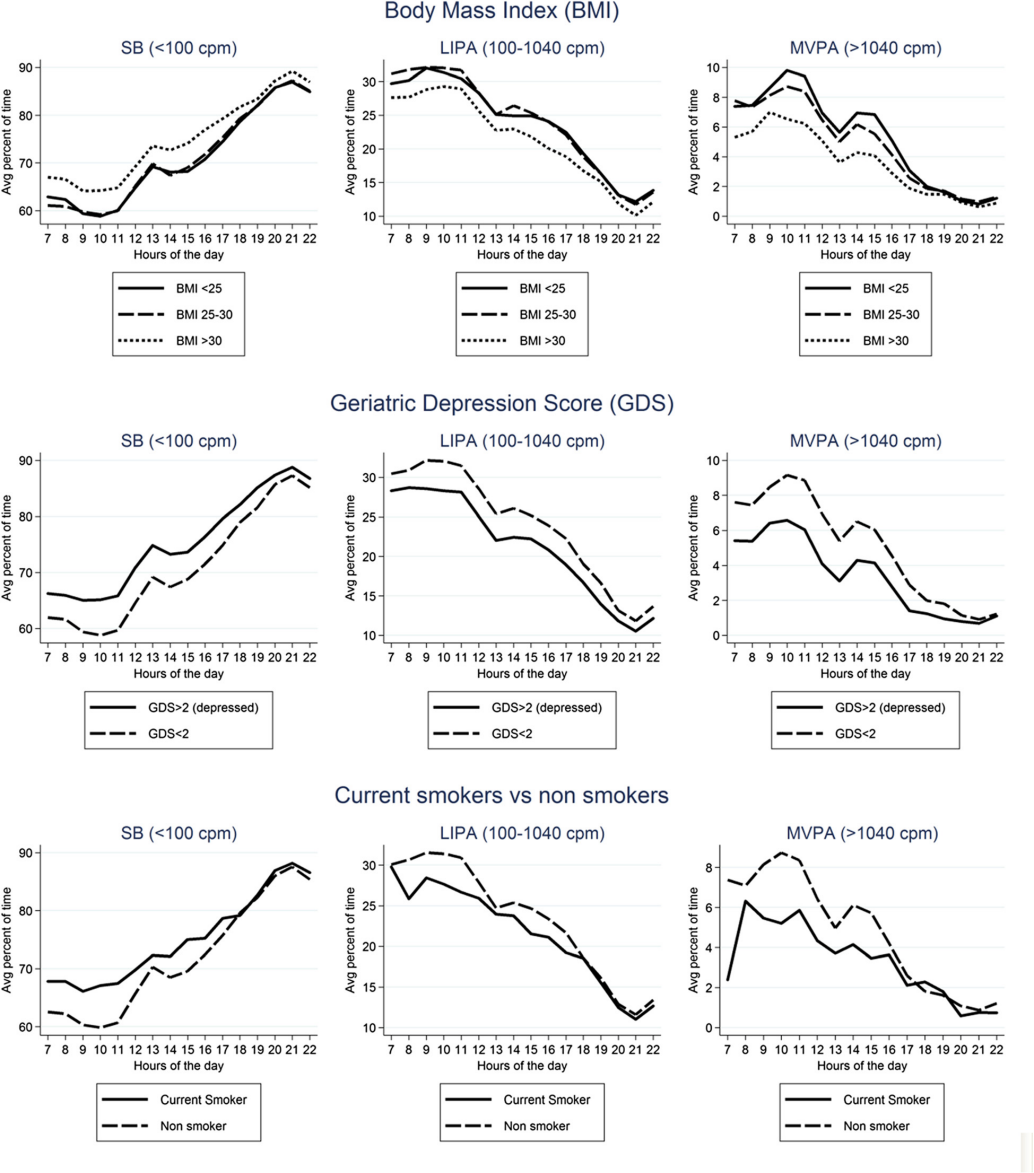
Plots from raw data: percentage of total wear time spent in SB, LIPA, and MVPA according to hour of day, stratified by BMI, depression, and smoking status

**Fig. 6:**
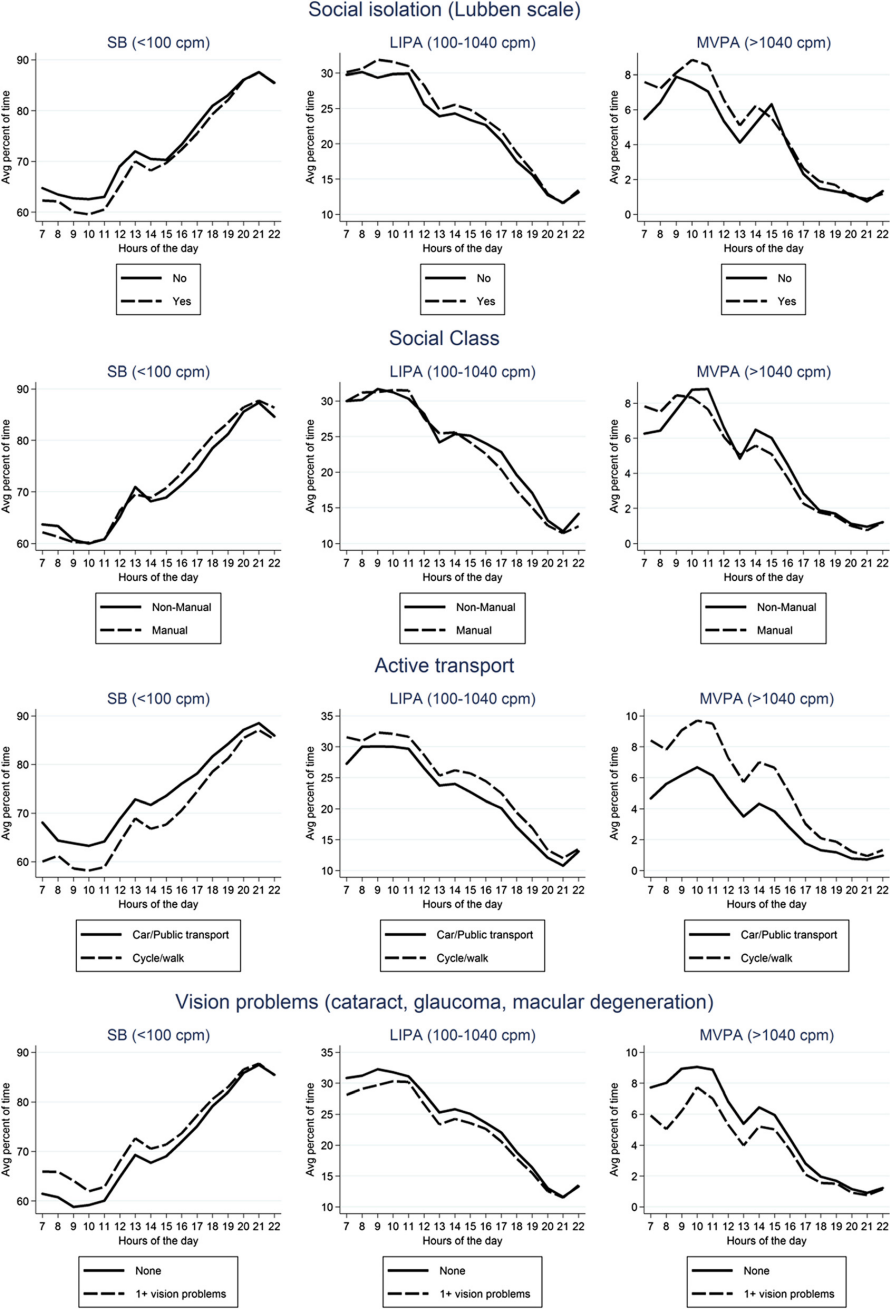
Plots from raw data: percentage of total wear time spent in SB, LIPA, and MVPA according to hour of day, stratified by social isolation, social class, use of active transport, season, and vision problems

**Fig. 7:**
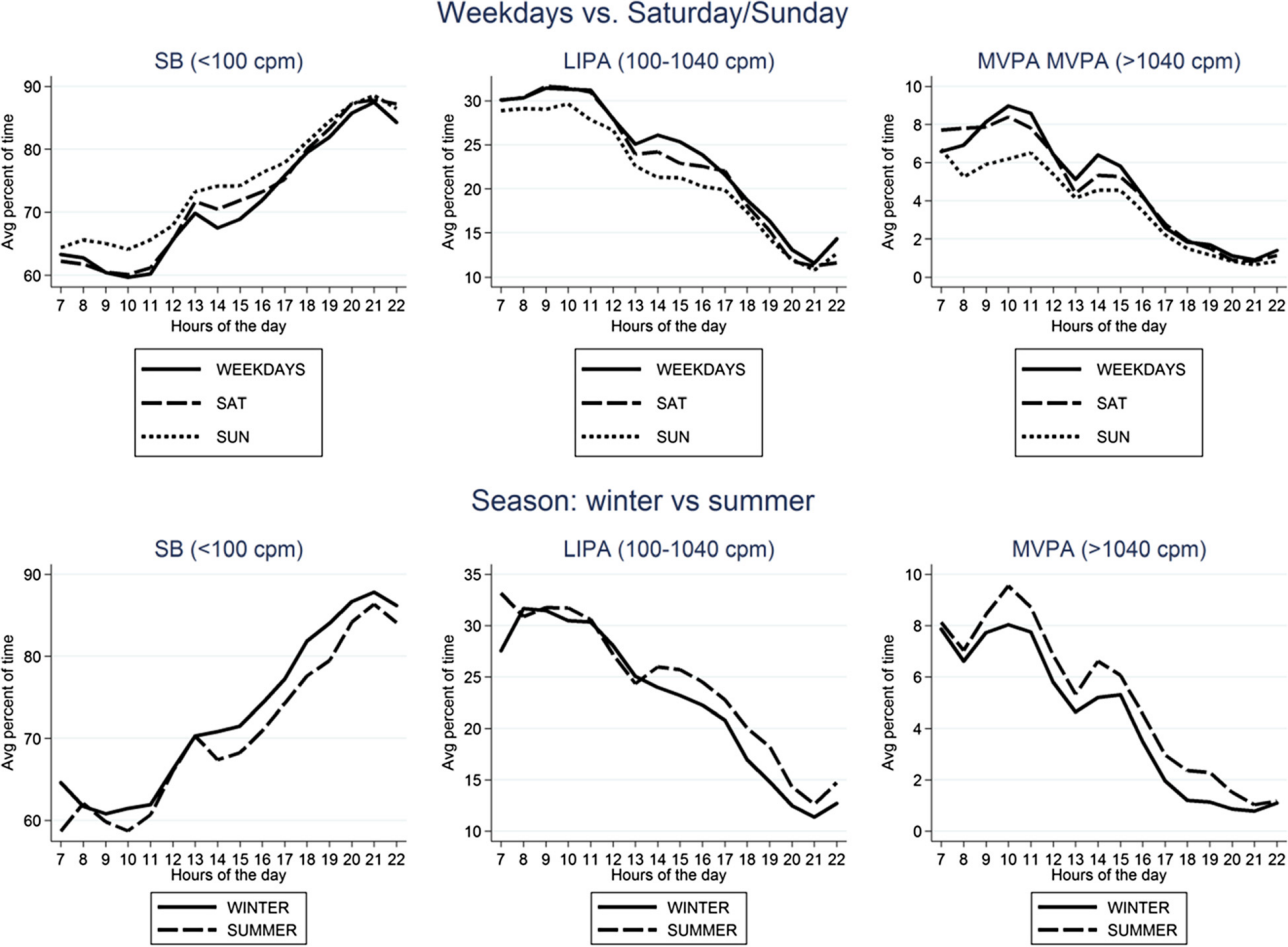
Plots from raw data: percentage of total wear time spent in SB, LIPA, and MVPA according to hour of day, stratified by day of the week and season

Univariable descriptive plots show hourly patterns of different intensities of physical activity stratified by age, mobility limitations, chronic diseases, BMI, geriatric depression score and smoking status (Figs. 4 and 5) and by social isolation, social class, active transport and vision problems, season (winter vs summer) and day of the week (Figs. 6 and 7). In most cases, the patterns of mean percentage of SB, LIPA and MVPA by hour of the day followed a consistent pattern of peaks and dips at the same time of day. Descriptive statistics on sedentary and MVPA bouts (plots not presented) showed consistent results with patterns in Figs. 4, 6, 7. 49.0 % of the sedentary bouts lasting ≥60 min over a valid week occurred in the evenings; most started between 8-9 pm (13.6 %) or 9-10 pm (14.0 %). Conversely, most (59.5 %) of MVPA bouts lasting ≥10 min over a valid week occurred in the morning and in particular when the peaks of MVPA were reported, at 10 am (15.9 %) and 11 am (16.4 %).

### Associations of social and demographic factors with PA and SB

Associations (main effects) of social and demographic factors with total time spent in physical activity and sedentary behaviour each day were estimated using multilevel models. The magnitude and significance of the associations did not differ greatly when adjusted for just one explanatory variable at a time or with further adjustments for all explanatory variables together (Table 2 for SB and LIPA and Table 3 for MVPA), hence the fully adjusted results are reported. The diurnal patterns estimated from the model were that average time spent in SB increased in the afternoon (+9 %) and evening (+21 %) when compared to morning (Table 2, Model 1). Conversely, time spent in LIPA decreased by -6 % and -16 % during afternoon and evening respectively (Table 2, Model 2). Additionally, percentage of time spent in SB each day was significantly higher and the percentage of the day in LIPA was significantly lower in the following groups; age ≥80 years, any mobility limitations, three or more chronic diseases, and obese. Total levels of SB were higher in the men who were depressed and did not use active transport, although LIPA did not vary by these characteristics. Neither LIPA nor SB differed by social class, presence of social isolation and vision problems.

**Table 2:** Adjusted associations between demographic and health factors and physical activity levels: percent of time spent in SB and LIPA^a^

	Model 1	Model 2
	Percent of time spent in SB (<100 CPM)	Percent of time spent in LIPA (100-1040 CPM)
	β (95 % CI)^b^	β (95 % CI)^b^
Part of the day (ref: Morning)		
Afternoon	9 (9,10)	-6 (-7,-6)
Evening	21 (21,22)	-16 (-16,-15)
Age categories (ref: age < 75 years old)		
75-79 years old	0.5 (-0.4,1.5)	-0.1(-0.9,0.7)
80+ years old	3.5 (2.5,4.5)	-2.3(-3.1,-1.4)
Mobility limitation (ref: no mobility limitations)		
slight mobility limitations	1.6 (0.6,2.6)	-1.1(-2.0,-0.3)
moderate/severe limitations or unable to do	3.3 (2.1,4.6)	-2.6(-3.6,-1.5)
Chronic conditions (ref: no chronic diseases)		
1-2 chronic diseases	0.7 (-0.1,1.5)	-0.6(-1.3,0.1)
3+ chronic diseases	2.6 (1.0,4.2)	-2.1(-3.4,-0.8)
Obese (ref: non-obese, BMI < 30)	1.4 (0.5,2.4)	-1.2(-2.0,-0.3)
Depressed (ref: not depressed)	0.9 (0.0,1.9)	-0.8(-1.6,0.1)
Current smoker (ref: non-smoker)	0.8 (-1.3,3.0)	-0.6(-2.5,1.2)
Use car/public transport (ref: cycle/walk)	1.0 (0.2,1.9)	-0.6(-1.3,0.1)
Social isolated (ref: not isolated)	0.2 (-0.8,1.2)	-0.2(-1.1,0.6)
Manual social class (ref: non manual)	0.2 (-0.5,1.0)	0.2(-0.5,0.9)
Vision problems (ref: none)	-0.2 (-0.6,1.1)	0.2(-0.6,0.9)

^a^Complete case analysis (n = 1329 in each model): men who met the inclusion criteria (Fig. 1) and who had at least two valid hours of wear time in each period of the day (morning 7:00-12:59, afternoon 13:00-18:59, evening 19:00-22:59). A valid hour is defined as an hour with ≥45 min of wear time

^b^β coefficient represents the difference in percent of time spent in SB (Model 1) and LIPA (Model 2) compared to the reference category of each explanatory variable. Models are multilevel linear regression models mutually adjusted for season of accelerometer wear, region of residence plus all explanatory variables listed in the table

**Table 3:** Adjusted Rate Ratios (RRs) for the percent of time spent in MVPA using two different cut offs (>1040 and >1951 counts per minute) according to demographic and health status variables^a^

	Model 1	Model 2
	Percent of time spent in MVPA (>1040 CPM)	Percent of time spent in MVPA (>1951 CPM)
	RR (95 % CI)^b^	RR (95 % CI)^b^
Part of the day (ref: Morning)		
Afternoon	0.57 (0.54,0.59)	0.50 (0.46,0.54)
Evening	0.17 (0.15,0.18)	0.11 (0.10,0.13)
Age categories (ref: age < 75 years old)		
75-79 years old	0.87 (0.79,0.96)	0.82 (0.71,0.95)
80+ years old	0.55 (0.50,0.61)	0.49 (0.42,0.57)
Mobility limitation (ref: no mobility limitations)		
slight mobility limitations	0.79 (0.71,0.89)	0.69 (0.59,0.81)
moderate/severe limitations or unable to do	0.50 (0.43,0.57)	0.33 (0.26,0.41)
Chronic conditions (ref: no chronic diseases)		
1-2 chronic diseases	0.91 (0.83,0.99)	0.91 (0.80,1.03)
3+ chronic diseases	0.66 (0.55,0.79)	0.65 (0.50,0.85)
Obese (ref: non-obese, BMI < 30)	0.83 (0.75,0.93)	0.72 (0.61,0.84)
Depressed (ref: not depressed)	0.88 (0.79,0.98)	0.92 (0.79,1.08)
Current smoker (ref: non-smoker)	0.76 (0.60,0.97)	0.85 (0.60,1.21)
Use car/public transport (ref: cycle/walk)	0.75 (0.68,0.82)	0.62 (0.55,0.72)
Social isolated (ref: not isolated)	0.97 (0.87,1.08)	1.11 (0.95,1.31)
Manual social class (ref: non manual)	0.93 (0.85,1.01)	0.98 (0.86,1.11)
Vision problems (ref: none)	0.96 (0.87,1.05)	1.00 (0.88,1.15)

^a^Complete case analysis (n = 1329 in each model): men who met the inclusion criteria (Fig. 1) and who had at least two valid hours of wear time in each period of the day (morning 7:00-12:59, afternoon 13:00-18:59, evening 19:00-22:59). A valid hour is defined as an hour with ≥45 min of wear time

^b^A Rate Ratio (RR) is a multiplicative factor. Compared to the reference category of each explanatory variable, any deviation from 1 indicates a change in percent of time spent in MVPA and a value < 1 indicates a decrease in MVPA (*e.g.* RR = 0.91 means a decrease in MVPA by a factor of 0.91 compared to the reference, that is about 10 %). Model 1 and 2 are negative binomial multilevel regression models mutually adjusted for season of accelerometer wear, region of residence plus all the explanatory variables in the table

The MVPA results were reported as RRs rather than beta coefficients, due to non-normality of the outcome distribution. The decline in MVPA was particularly marked over the course of the day; compared to the morning, levels of MVPA (>1040 CPM, Table 3 Model 1) declined substantially in the afternoon (RR = 0.75, 95 % CI 0.54-0.59) and in the evening (RR = 0.17, 0.15-0.18). Additionally, men with moderate or more severe mobility limitations compared to the reference category (no mobility limitations) had a RR of 0.50 (95 % CI 0.43, 0.57) for MVPA indicating that men with mobility limitations were half as likely to spend time doing MVPA compared to people with no limitations. Moreover, men who were older, did not use active transport, were obese, depressed, had more chronic health conditions, and were smokers had lower levels of MVPA.

Similar associations were seen when these analyses were repeated with a higher cut point (1952 CPM) to define MVPA (Table 3, Model 2). The largest RRs (risks of having low MVPA levels) were for being over 80 compared to less than 75 years, for the category “moderate/severe limitations or unable to do” if compared with no mobility limitations and use of active transport versus car/public transport. However, when using the 1952 CPM cut-point, MVPA level no longer differed by depression or smoking status.

### Factors associated with modified diurnal patterns of PA and SB

Four factors were significantly associated with each of SB, LIPA and MVPA levels: age, mobility limitations, chronic diseases, and BMI. The effects of older age, obesity, mobility limitations and chronic diseases on LIPA, MVPA and SB appeared to be more marked in the morning than in the afternoon and evening, independent of the lower overall levels of PA observed in these subgroups. Interaction tests were performed to establish whether these associations differed by period of the day. The tests for interaction (Wald tests) were all statistically significant (*p* < 0.05 for age, chronic conditions and BMI and *p* <0.001 for mobility limitations).

## Discussion

This study investigated the diurnal variations in accelerometer-measured PA and SB levels in a large sample of older British men. The analyses demonstrated that the total amount of physical activity (steps and CPM) was highest in the morning but then decreased during the day, except for a small increase at 2-3 pm. LIPA and MVPA showed a similar pattern. Conversely, SB levels were lowest in the morning and increased throughout the day, peaking at 9 pm (88 %). Commonly reported activities in the morning were shopping, gardening, housework and DIY.

We also examined diurnal variations of SB and PA by demographic factors and health status. Men who were older, had mobility limitations, more chronic health conditions and were obese tended to spend more time in SB and were less physically active. Moreover, men who did not use active transport, who were depressed and smoked cigarettes spent less time in MVPA, but those factors did not affect SB or LIPA. Age, mobility limitations, chronic conditions and obesity influenced LIPA, MVPA and SB levels in the morning more than in the afternoon and evening. The findings showed an attenuation of the diurnal pattern among less active subgroups (*e.g.* older and more infirm men). This reflects their diminished ability to maintain relatively high intensity physical activity during the morning, and this is not simply related to the generally low PA level typical of these subgroups. This information is important for policy and practice because there is scope to extend the tendency for existing activity bouts during the morning and early afternoon and also to increase activity levels later in the afternoon. Our analysis of whether or not the effects of specific health and social variables on PA levels varied by time of day offers unique new insights, as previous studies of older adults have not considered this question.

### Comparisons with other studies

To date there has been little work using hourly accelerometer data to examine diurnal physical activity patterns among older adults. Our results showing that physical activity peaks in the morning are consistent with recent studies [2, 4, 6–8, 11], although our study extends the literature by investigating more intensities of activity (SB, light PA and MVPA) and bouts of activities. In our study the overall PA levels (measured as CPM) over the course of the day were similar to other smaller studies using the same measurement device [4, 6–8, 11]. We examined PA patterns between 7.00 am and 10.59 pm and a similar period (6 am-10 pm or 7 am-9 pm) was analysed in other studies due to sparse data in early morning and late evening [2]. In line with our findings, a study of 38 healthy active adults (mean age 70 years) reported significantly fewer minutes of MVPA in the evening than in the morning or afternoon [11] and that longer bouts of activity occurred in the morning (6 am-12 pm) more often than afternoon or evening. The AGES-II study of 579 adults aged 73–98 from Iceland reported that the majority of PA occurred between 8 am and 4 pm on an average day [4], which fits with our findings. In line with our study, they also reported that sedentary time was similar across all age groups, except for the oldest age group (>85 years old) who were the most sedentary and PA levels declined with increasing age and BMI, but other social and health factors were not taken into account. Our findings about age modifying the daily patterns in physical activity fit with data from the AGES-II study [4] and from the Baltimore Longitudinal study of Ageing [6], which also found that older age groups had a steeper decline in in PA levels over the course of the day. Our finding that activity levels were lower in the mornings in obese than normal weight men mirrors data from a study of Canadian adults aged 20-79 years [2]. To our knowledge other studies have not investigated how presence of chronic conditions and mobility limitations affect the diurnal patterns of physical activity and sedentary behaviour in older adults.

### Strengths and limitations

This study investigates how hourly levels of objectively measured SB, LIPA and MVPA vary over the course of the day and how daily activity patterns are modified by a wide range of demographic and health characteristics. It is particularly important to investigate LIPA in population based samples of older adults, because of the high proportion of time spent in light activity. Our findings about the correlates of LIPA offer a new contribution to the ongoing debate about whether and how the PA guidelines should include recommendations on LIPA as well as MVPA [27, 28].

This study benefits from using a large scale population-based cohort of free-living older men rather than a special *at risk* population, which should increase generalizability. The response rate achieved in this study is comparable with other studies on objective measurements of daily physical activity patterns [4]. Men who did not accept our invitation were about two years older and had higher BMI measured 10 years earlier; implying that overall PA (*e.g.* total counts or number of steps) might be lower in the general population. Our study is however limited by studying only white European men, who, based on existing literature, would be expected to have higher levels of PA, particularly MVPA, compared with women [9]. Therefore our results may not be generalizable to older women or ethnic minority populations [29]. Our study did not report detailed information on mode of activity, which was self-reported only during the first three days of accelerometer wearing. However, the importance of this information is recognized and future studies could investigate further the particular types of activities carried out during the highest and lowest peaks of activity [30]. A further area for future study would be the assessment of seasonal patterns of PA and SB (although these were not an objective of this study). Future analysis is needed to determine whether or not seasonal variations in PA and SB are observed even after adjustment for confounding of weather variables (*e.g.* temperature or sunshine duration). We investigated whether diurnal patterns in PA and SB were modified by season (winter vs. summer) and day of the week (Sunday vs Saturday or Monday-Friday). Whilst PA levels are generally lower (and SB levels higher) in winter and on Sundays, but we did not find evidence of effect modification on diurnal patterns.

### Implications

The marked variations in PA occurring on a within-day basis provide information which could be helpful in planning interventions to increase PA levels. Older adults do most of their MVPA and light activity during the morning. Thus, one possible strategy for interventions aiming to increase these intensities of activity would be either to focus on the morning when people are already active and when variability in activity levels are greatest, aiming to increase the intensity or duration of existing physical activity bouts. Alternatively, interventions could focus on the afternoon period, aiming to stimulate physical activity of comparable intensity to that occurring in the morning. It is unlikely that low levels of activity in the evening can be changed, particularly in the winter months if it is dark in the late afternoons and evenings. Indeed, the combination of darkness and visual problems have been previously investigated as potential causes of falls [31]. Likewise with sedentary behaviours, our findings suggest that the period in the late afternoon and early evenings are periods with high levels of SB and when bouts of SB are likely to be longest, so it may be particularly valuable to focus on efforts to break up long sedentary bouts at these times of day. Our investigation showed that age and health status affected these diurnal patterns suggesting that PA policies might be targeted by sub-groups. Among older and disabled men, lower levels of MVPA were observed in morning and afternoon than in younger healthy men, the morning peak was more reduced than the afternoon peak, suggesting that with increasing age, the higher morning peak in moderate to vigorous activity may be particularly difficult to maintain. Longitudinal analyses could offer additional insights and determine if there are independent effects on health of MVPA or SB at different times of the day.

## Conclusions

This study provides detailed data about diurnal patterns in habitual physical activity levels in free-living older men which can inform the development of effective programmes to encourage older men to be physically active. This study highlights that especially among men over 80 years old, who are obese, with multiple chronic diseases or with mobility limitations there are particular opportunities to maintain or enhance existing activity bouts during the morning and early afternoon and to reduce the duration of SB periods in the afternoon and evening hours.

## Abbreviations

CPM: Counts per minute
PA: Physical activity
LIPA: Light physical activity
MVPA: Moderate to vigorous physical activity
SB: Sedentary behaviour
MET: Metabolic equivalent of task
RR: Rate ratio
